# Quercetagetin-rich flavonoids from marigold induce apoptosis inhibit metastasis of non-small cell lung cancer via the p53/p21 signaling pathway

**DOI:** 10.3389/fonc.2026.1813351

**Published:** 2026-06-26

**Authors:** Zhihuan Dong, Xinying Cheng, Yunhe Lian, Di Wu, Kaihui Liu, Xue Feng, Qiang Xue, Qingliang Chen, Limin Wang

**Affiliations:** 1 Clinical School of Thoracic, Tianjin Medical University, Tianjin, China; 2 Department of Cardiac Surgery, Handan First Hospital, Handan, Hebei, China; 3 Hebei Chenguang Detection Technology Services Co., Ltd, Handan, Hebei, China; 4 Chenguang Bio-technology Group Co., Ltd, Handan, Hebei, China; 5 Department of Cardiovascular Surgery, Tianjin Chest Hospital, Tianjin, China; 6 School of Medicine, Hebei University of Engineering, Handan, Hebei, China

**Keywords:** apoptosis, metastasis, NSCLC, p53/p21 signaling pathway, QG-MF, Tagetes erecta L.

## Abstract

Non-small cell lung cancer (NSCLC) remains a leading cause of cancer-related mortality worldwide, with limited therapeutic efficacy and severe side effects associated with conventional chemotherapy, creating an urgent need for safe and effective natural anti-tumor agents. Quercetagetin-rich marigold flavonoids (QG-MF), isolated from the lutein-extracted inflorescence residues of *Tagetes erecta* L. (marigold), have been identified as bioactive polyphenols with potential anti-cancer properties, yet their anti-NSCLC activity and underlying molecular mechanisms remain elusive.

**Aim of the study:**

This study aimed to investigate the *in vitro* and *in vivo* inhibitory effects of QG-MF on NSCLC.

**Methods:**

*In vitro* experiments were performed on A549 and H661 NSCLC cell lines: cell proliferation was detected by CCK-8 assay; cell migration and invasion were evaluated via wound healing and Transwell assays, respectively; cell apoptosis and cycle distribution were analyzed by flow cytometry; the expression levels of p53/p21 signaling pathway-related proteins were determined by Western blot. *In vivo* anti-tumor efficacy of QG-MF was assessed using A549 NSCLC xenograft mouse models, with tumor volume and weight measured to evaluate tumor growth inhibition.

**Results:**

QG-MF significantly suppressed proliferation, migration and invasion, and induced apoptosis as well as cell cycle arrest in A549 and H661 cells in a concentration-dependent manner. Mechanistically, QG-MF upregulated the expression of p53, p21, Bax and caspase-3, and downregulated Bcl-2 in NSCLC cells. Furthermore, siRNA-mediated p53 silencing effectively abrogated the anti-proliferative and pro-apoptotic effects of QG-MF on NSCLC cells. *In vivo*, QG-MF markedly inhibited the growth of A549 xenograft tumors in nude mice without obvious systemic toxicity.

**Conclusion:**

Our findings demonstrate that QG-MF exerts potent anti-NSCLC activity by inducing apoptosis and inhibiting proliferation/metastasis via the p53/p21 signaling pathway.

## 1 Introduction

NSCLC, which accounts for approximately 85%-90% of all lung cancer cases, is one of the most common types of cancer worldwide (1). The treatment outcomes for lung cancer are unsatisfactory, with the overall five-year survival rate below 15% for patients with locally advanced NSCLC and below 5% for those with advanced-stage disease (2). Because NSCLC lacks noticeable early symptoms, about 60% of patients are diagnosed with distant metastasis at initial presentation, and this is a primary reason for its poor prognosis (3). Metastasis is currently the leading cause of treatment failure in NSCLC, contributing to over 90% of cancer-related deaths (4). Although chemotherapy remains the first-line treatment for NSCLC, it is generally associated with issues such as drug resistance and intolerable side effects (5). Therefore, there is an urgent need to develop a drug that can inhibit tumor proliferation and invasive metastasis.

Natural products derived from medicinal plants have long been a valuable source for anti-tumor drug discovery, owing to their abundant structural diversity, prominent biological activities and low toxicity (6–8). Flavonoids, as a large class of polyphenolic secondary metabolites from medicinal plants, have been well-documented to exert multiple anti-tumor effects, including the inhibition of tumor cell proliferation, induction of apoptosis, suppression of angiogenesis, and reversal of drug resistance (9). Among these, quercetin and luteolin are well-known flavonoids with reported anti-cancer activities; however, their clinical application is often limited by poor bioavailability. Furthermore, the high-purity extraction of these compounds from their primary plant sources can be economically challenging (10, 11).

*Tagetes erecta* L. (marigold), a widely cultivated ornamental and medicinal herb, has a long history of ethnomedicinal use in China and other countries; in traditional Chinese medicine (TCM), it is traditionally prescribed for the treatment of inflammatory disorders due to its heat-clearing and detoxifying properties (12). Quercetagetin, a major flavonol component isolated from *Tagetes erecta* L. extracts, is a highly bioactive compound with antioxidant, anti-inflammatory, antibacterial, and lipid-lowering properties (13). Accumulating evidence has demonstrated that quercetagetin exerts anti-tumor effects against various malignant tumors, such as breast cancer (14), nasopharyngeal cancers (15), and glioblastoma (16).

Notably, unlike most flavonoid-based therapies that rely on directly harvested plant materials or synthetic derivatives, the present study utilizes the inflorescence residue of T. erecta L.—a major agricultural byproduct generated during industrial lutein extraction. The valorization of such plant processing residues into high-value bioactive products not only avoids resource waste and environmental pollution but also aligns with the global trend of sustainable development. This unique source distinguishes QG-MF from conventional flavonoid preparations, as it offers a cost-effective, eco-friendly, and scalable strategy. Previous work by the current team successfully extracted and identified Quercetagetin-rich total flavonoids (QG-MF) from the lutein-extracted inflorescence residues of T. erecta L., with a total flavonoid purity of 94% and quercetagetin accounting for 85% of the total flavonoid content (17, 18).However, the anti-tumor activity of this residue-derived QG-MF against NSCLC, as well as its underlying molecular mechanisms, have not yet been elucidated.

In the present study, we aimed to investigate the *in vitro* inhibitory effects of QG-MF on NSCLC cell proliferation, invasion, and metastasis, and to clarify the potential regulatory role of the p53/p21 signaling pathway in mediating these anti-tumor effects. Furthermore, we evaluated the *in vivo* anti-tumor efficacy of QG-MF using a NSCLC xenograft mouse model to validate its therapeutic potential. This work not only provides experimental evidence for the ethnopharmacological application of T. erecta L. but also lays a scientific foundation for the high-value utilization of industrial marigold lutein residues, and identifies QG-MF as a promising natural candidate for the development of novel anti-NSCLC agents.

## 2 Materials and methods

### 2.1 Reagents

Cisplatin (CDDP) was purchased from Sigma-Aldrich Inc. (232120, Sigma-Aldrich, St Louis, MO). Antibodies used for western blotting were mouse anti-TP53 (TA502870M, OriGene, Technologies, Inc., Rockville, MD), rabbit anti-p21 (TA327053, OriGene, Technologies, Inc.), mouse anti-Bcl-2 (TA806591M, OriGene, Technologies, Inc.), rabbit anti-Bax (5023, Cell Signaling Technology), rabbit anti-caspase-3 (TA383876mM, OriGene, Technologies, Inc.), rabbit anti-GAPDH (5174, Cell Signaling Technology). The secondary antibodies coupled to HRP were purchased from ZSGB-BIO (Beijing, China).

### 2.2 Preparation of quercetagetin-rich flavonoids

Quercetagetin-rich flavonoids (QG-MF) were provided by our team (Chenguang Biotechnology Group Co., Ltd., Handan, Hebei, China; batch No. Y3-2720-19102411), with a total flavonoid purity of 94% and quercetagetin constituting 85% of the total content. Its extraction and component identification were described in a prior study (17),authored by some co-authors of this work. For *in vitro* and *in vivo* assays, QG-MF powder was dissolved in DMSO via 5 minutes of ultrasonication to prepare a stock solution with a concentration of 300 mg/mL. This stock solution was freshly prepared prior to each use without storage, and further diluted more than 1000-fold to obtain the working solution.

### 2.3 Cell culture

Human lung cancer cell lines A549 and H661 were purchased from Shanghai Cell Bank, Chinese Academy of Sciences. Cells were grown and maintained in DMEM medium (GIBCO BRL, Grand Island, NY, USA), supplemented with 10% fetal bovine serum, at 37 °C, 5% CO2.

### 2.4 Cell proliferation assay

Cells were plated in a 96-well plate at a density of 6 × 10^3^ cells per well. The experiment was performed in triplicate. At 24 h post-seeding, QG-MF was added to the culture medium at concentrations ranging from 0 to 250 μg/mL. The cells were then cultured for an additional 48 hours. Cells cultured in fresh medium were used as the control group. Cell proliferation was determined using the Cell Counting Kit-8 (CCK-8; Solarbio Science and Technology Co., Ltd., Beijing, China) according to the manufacturer’s instructions.

### 2.5 Wound healing assay

Cell migration was evaluated with a wound healing assay and the experiment was performed in triplicate. Cells were seeded in 6-well plates at a density of 2.5 × 10^5^ cells per well. The treatment group was treated with QG-MF at concentrations of 15 or 20 μg/mL for 24 h, while the control group was cultured in fresh medium. A scratch wound was created in the cell monolayer using a sterile pipette tip when the cells reached 95% confluency. After scratching, the detached and floating cells were thoroughly removed by gentle washing with PBS to eliminate potential interfering factors. Subsequently, cells were incubated in routine complete medium for the following 24 h and 48 h. The wounds were imaged at 0, 24, and 48 hours under a light microscope (Nikon, Tokyo, Japan) at 40x magnification to measure the migration distance of the cells.

### 2.6 Cell invasion assay

Cell invasion was assessed using a Matrigel-coated trans-well chamber (24-well format, 8-μm pores; Costar) and all experiments were performed in triplicate. Briefly, cells were pretreated with QG-MF at concentrations of 15 μg/mL and 20 μg/mL, while cells in the control group were cultured in normal DMEM medium for 24 h. Thereafter, 1 × 10^4^ pretreated cells were seeded into the upper chamber with serum-free medium. The lower chamber was filled with DMEM containing 10% FBS. After being cultured for 48 h in a 37°C, 5% CO_2_ atmosphere, non-invading cells on the upper surface of the membrane were removed with a cotton swab; invaded cells on the lower surface of the membrane were stained with 1% crystal violet and counted in 10 random fields per well under a microscope at 200x magnification.

### 2.7 RNA interference and transfection

Lung cancer cells were plated in 6-well plates at a density of 1.5 × 10^5^ cells/well and transfected with either p53-targeting siRNA or negative control (NC) siRNA at a final concentration of 25 nM using KeygenMAX 3000 transfection reagent (KeyGEN BioTECH, China) according to the manufacturer’s instructions. All transfection experiments were performed in triplicate. The sequence of p53-targeting siRNA (APEXBIO, USA) was as follows: sense, 5’-GAAAUUUGCGUGUGGAGUATT-3’; antisense, 5’-UACUCCACACGCAAAUUUCTT-3’. The sequence of NC siRNA (APEXBIO, USA) was listed below: sense, 5’-UUCUCCGAACGUGUCACGUTT-3’; antisense, 5’-ACGUGACACGUUCGGAGAATT-3’. Cells were continuously incubated for 48 h after transfection prior to subsequent experimental analysis.

### 2.8 Apoptosis and cell cycle analysis

Cells were seeded in six-well plates (at 1.5 × 10^5^ cells per well) and cultured overnight. The cells were treated with QG-MF at concentrations of 15, 20, or 25 μg/mL for 48 h. Subsequently, the cells were harvested, centrifuged at 1500 rpm for 5 min, and washed with PBS. For cell cycle analysis, the harvested cells were fixed in ice-cold 75% ethanol and stored at −20 °C overnight. The fixed cells were then centrifuged (1500 rpm, 5 min, 4 °C), washed twice with cold PBS. After the final wash, the cell pellet was resuspended in 500 µL of propidium iodide/RNase staining buffer (F10797, Thermo Fisher Scientific) and incubated for 30 min at room temperature in the dark. Finally, apoptosis analysis was performed on a FACSAria flow cytometer (Becton Dickenson, San Jose, CA, USA).

Apoptosis was analyzed using a FITC Annexin V Apoptosis Detection Kit (556547, BD Biosciences). The cells were treated with QG-MF at concentrations of 15, 20, or 25 μg/mL for 48 h. After treatment, cells were harvested, washed twice with cold PBS, and resuspended in 1X Binding Buffer at a density of 1 × 10^6^ cells/mL. Then, 100 µL of the cell suspension was incubated with 5 µL of FITC Annexin V and 5 µL of PI in the dark at room temperature for 15 min. After the incubation, 400 µL of 1X Binding Buffer was added, and the samples were analyzed by flow cytometry.

### 2.9 Proteomic data analysis

Proteomic analysis was conducted by Shanghai Aipudikang Biotechnology Co., Ltd. (Shanghai, China). Raw mass spectrometry data were analyzed on the iProteome cloud platform with a custom spectral library. Search parameters: trypsin digestion; fixed modification: carbamidomethyl (C); variable modifications: oxidation (M) and protein N-terminal acetylation; maximum missed cleavages: 2; peptide mass tolerance: 20 ppm; fragment mass tolerance: 0.05 Da. Proteins were quantified via label-free DIA, and abundance was normalized using the fraction of total (FOT) method.

Differentially expressed proteins (DEPs) were screened by unpaired two-tailed Student’s t-test. Proteins with |log_2_(fold change)| ≥ 1.0 and a p-value < 0.05 were defined as DEPs.

Gene Ontology (GO) enrichment was performed based on annotations from NCBI, UniProt and GO databases, with InterProScan supplemented for species-specific information. All identified proteins served as the background. Fisher’s exact test was used to detect enriched GO terms, and the Benjamini–Hochberg method was applied for multiple testing correction. terms with a p-value < 0.05 were considered significantly enriched.

### 2.10 Western blotting

Cells were seeded in 6-well plates. The treatment group was treated with QG-MF at concentrations of 15, 20, or 25 μg/mL for 48h, while the control group was cultured in fresh medium. Then, the cells were harvested and lysed with RIPA buffer containing protease inhibitor (Sigma-Aldrich). Total protein was quantified using the Pierce™ BCA Protein Assay Kit (23225, Thermo Scientific, MA, USA). Equal amounts of protein were then separated by SDS-PAGE and transferred onto a nitrocellulose membrane (Millipore, Bedford, MA, USA). The membranes were blocked with 5% non-fat milk in Tris-buffered saline containing 0.5% Tween-20 (TBS-T) for 2 h at room temperature and then probed with primary antibodies against p53, p21, Bcl-2, Bax and caspase-3 (diluted in TBS-T containing 5% BSA) at 4 °C overnight. After washing three times with TBS-T, the membranes were probed with HRP-conjugated secondary antibodies for 2 h at room temperature. The protein bands were visualized using a chemiluminescent HRP substrate (Millipore, Bedford, MA, USA).

### 2.11 Mouse tumor models

Female BALB/c nude mice (4–5 weeks old) were purchased from Sibefu Biotechnology Co., Ltd. (Beijing, China). All xenograft experiments were performed in accordance with the National Institutes of Health (NIH) Guide for the Care and Use of Laboratory Animals, and were approved by the Experimental Animal Ethics Committee of Hebei Chenguang Testing Technology Service Co., Ltd. (Approval No.: JC-LL-W25003). Each treatment group consisted of 5 mice. Xenograft tumors were established by subcutaneously injecting 3 × 10^6^ A549 cells suspended in 100 μL PBS into the right lower flank of each mouse. When the tumor volumes reached approximately 60 mm^3^, the tumor-bearing mice were randomly assigned into three groups (n=5 per group): (1) the control group, (2) the QG-MF group, and (3) the CDDP group. The QG-MF group received QG-MF (360 mg/kg) orally by gavage once daily.This dose was determined based on relevant literature on flavonoid anti-tumor research (19)and preliminary dose screening with 250 mg/kg and 360 mg/kg; 360 mg/kg was selected for superior efficacy. The CDDP group received cisplatin (2 mg/kg) via intraperitoneal (i.p.) injection every two days. The control group was orally administered with an equivalent volume of normal saline following the same schedule as the QG-MF group.Tumor dimensions were measured every 3 days, and tumor volume was calculated as: volume (mm3) = d2 × D/2, where the d and D were the shortest and the longest diameters. The experiment had been performed for 6 weeks. Tumor tissues were dissected and used for protein extraction. Nude mice were euthanized by cervical dislocation without prior anesthesia to avoid potential confounding effects of anesthetics on the P53/P21 signaling pathway, which is highly sensitive to cellular stress, and all efforts were made to minimize animal suffering.

### 2.12 Statistical analysis

Data are presented as the mean ± SD. Statistical comparisons between two groups were performed using Student’s t-test. Comparisons among three or more groups were performed using one-way ANOVA, followed by Dunnett’s *post hoc* test for multiple comparisons against a control group. All analyses were conducted using SPSS 20.0. A p-value of less than 0.05 was considered statistically significant.

## 3 Results

### 3.1 QG-MF inhibited the growth, migration and invasion of lung cancer cells

**Figure 1:**
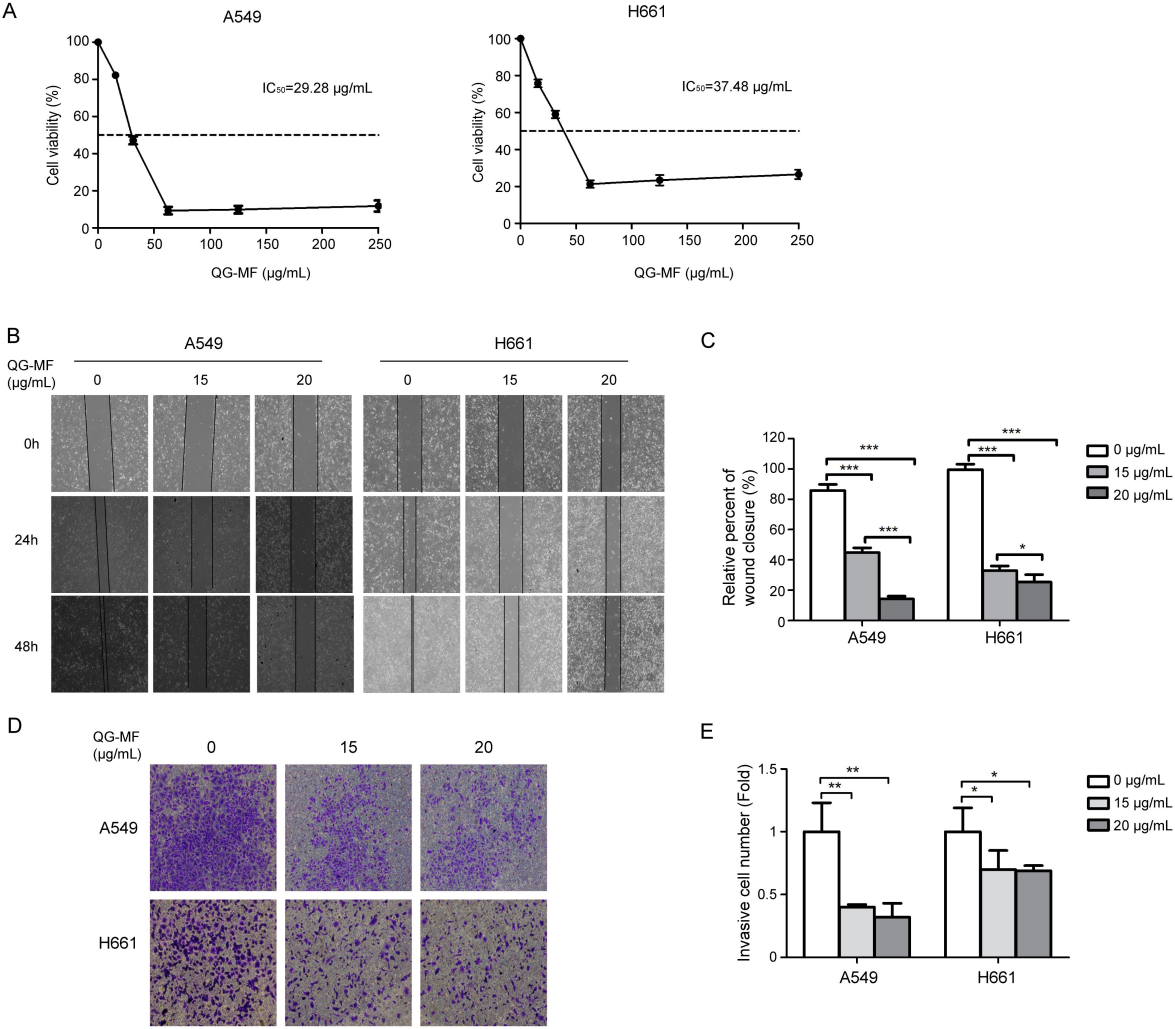
QG-MF inhibits the growth, migration and invasion of lung cancer cells. **(A)** Cell lines were treated with various concentrations of QG-MF (0–250 μg/mL) for 48 hours. The IC_50_ values were 29.28 μg/mL for A549 cells and 37.48 μg/mL for H661 cells. **(B)** Cell migration was assessed by wound healing assay after 48 h of treatment with QG-MF. Representative images (40×) are shown. **(C)** Quantitative analysis of wound closure. Data are expressed as the relative wound healing rate compared to the control group. **(D)** Cell invasion was evaluated by Transwell assay after 48 h of treatment with QG-MF. Representative images (200×) are shown. **(E)** Quantitative analysis of invasive cell numbers from **(C)**. Data are presented as the mean ± SD from three independent experiments. *P < 0.05, **P < 0.01, ***P < 0.001 vs. control group.

A549 (adenocarcinoma) and H661 (large cell carcinoma) cell lines were selected to represent distinct NSCLC pathological subtypes. To investigate the effect of QG-MF on lung cancer cell viability, both cell lines were treated with various concentrations of QG-MF (0–250 μg/mL) for 48 hours. Cell viability was then assessed using the CCK-8 assay. The cell viability gradually decreased with the increase of concentrations. The IC_50_ values were calculated to be 29.28 μg/mL for A549 cells and 37.48 μg/mL for H661 cells (**Figure 1A**). These findings indicate that QG-MF effectively inhibits the viability and proliferation of lung cancer cells.

To investigate the effect of QG-MF on cell migration, we treated A549 and H661 cells with QG-MF at concentrations of 15 or 20 μg/mL for 48 h, using DMEM as a vehicle control. A wound healing assay was then performed. The wound closure rates for A549 and H661 cells in the control group were 85.7% and 98.5%, respectively. QG-MF treatment significantly reduced the wound closure rates to 44.4% and 14.3% in A549 cells, and to 33.3% and 25.0% in H661 cells, at 15 and 20 μg/mL, respectively (**Figures 1B, C**).

We further performed Transwell assays to quantitatively validate the inhibitory effect of QG-MF on cell invasion. Consistent with the wound healing results, Transwell assays showed that QG-MF markedly suppressed the migration and invasion abilities of lung cancer cells in a dose-dependent manner (**Figures 1D, E**). Quantitative statistical analysis revealed that the relative invasive cell numbers of A549 cells were reduced to 0.40 and 0.32-fold of the control after treatment with 15 μg/mL and 20 μg/mL QG-MF, respectively. For H661 cells, the relative invasion rates decreased to 0.70 and 0.69-fold of the control under the corresponding treatments.

Taken together, our data indicate that QG-MF significantly inhibits the migratory and invasive capabilities of lung cancer cells.

### 3.2 QG-MF induced cell cycle-arrest and apoptosis in lung cancer cells

**Figure 2:**
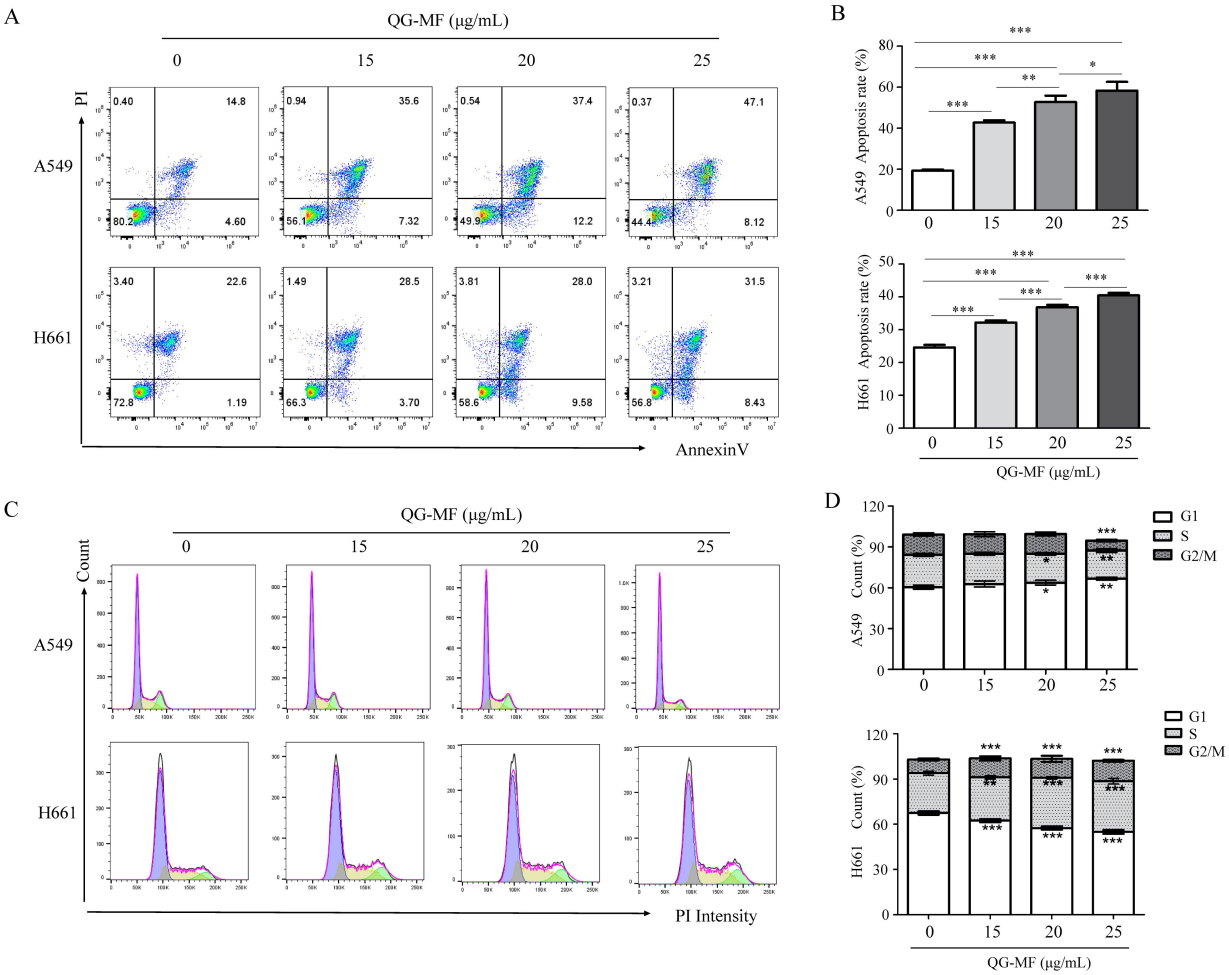
Effects of QG-MF on apoptosis and cell cycle on lung cancer cells. **(A)** Analysis of apoptosis by Annexin V-FITC/PI staining after treatment with the indicated doses of QG-MF for 48 h. **(B)** Quantification of total apoptotic cells (early + late apoptosis) from **(A)**. Data are presented as the mean ± SD (n = 3). *p < 0.05, **p < 0.01, ***p < 0.001 vs. control group. **(C)** Cell cycle distribution analyzed by PI staining and flow cytometry after QG-MF treatment for 48 h. **(D)** Quantitative analysis of the cell cycle from **(C)**. Data are presented as the mean percentage of cells in each phase ± SD (n = 3). *p < 0.05, **p < 0.01, ***p < 0.001 vs. control group for the same phase.

To investigate the pro-apoptotic effect of QG-MF, apoptosis was assessed using an Annexin V-FITC/PI staining kit followed by flow cytometry. The results revealed that QG-MF treatment significantly induced apoptosis in both cell lines in a dose-dependent manner. After 48 h of exposure, the apoptotic rates (including early and late apoptosis) in A549 cells increased to 42.79%, 52.75%, and 58.28% at concentrations of 15, 20, and 25 μg/mL, respectively. Similarly, the apoptotic rates in H661 cells reached 32.18%, 36.84%, and 40.48% at the corresponding concentrations (**Figures 2A, B**). In contrast, the control groups did not show significant apoptosis. These results demonstrate that QG-MF effectively induces apoptosis in lung cancer cells.

The cell cycle is critically involved in tumorigenesis and cancer progression. To investigate the effect of QG-MF on cell cycle distribution and determine whether this effect is dose-dependent, A549 and H661 cells were treated with various concentrations (15, 20, 25 μg/mL) of QG-MF. As shown in **Figure 2C**, QG-MF treatment induced cell cycle arrest in a dose-dependent manner. Specifically, it increased the proportion of A549 cells in the G0/G1 phase and the proportion of H661 cells in the S phase, compared to the control group (**Figure 2D**). These results indicate that QG-MF induces cell cycle arrest in lung cancer cells, thereby disrupting normal cell cycle progression.

### 3.3 QG-MF up-regulated the expression of p53 in lung cancer cells

**Figure 3:**
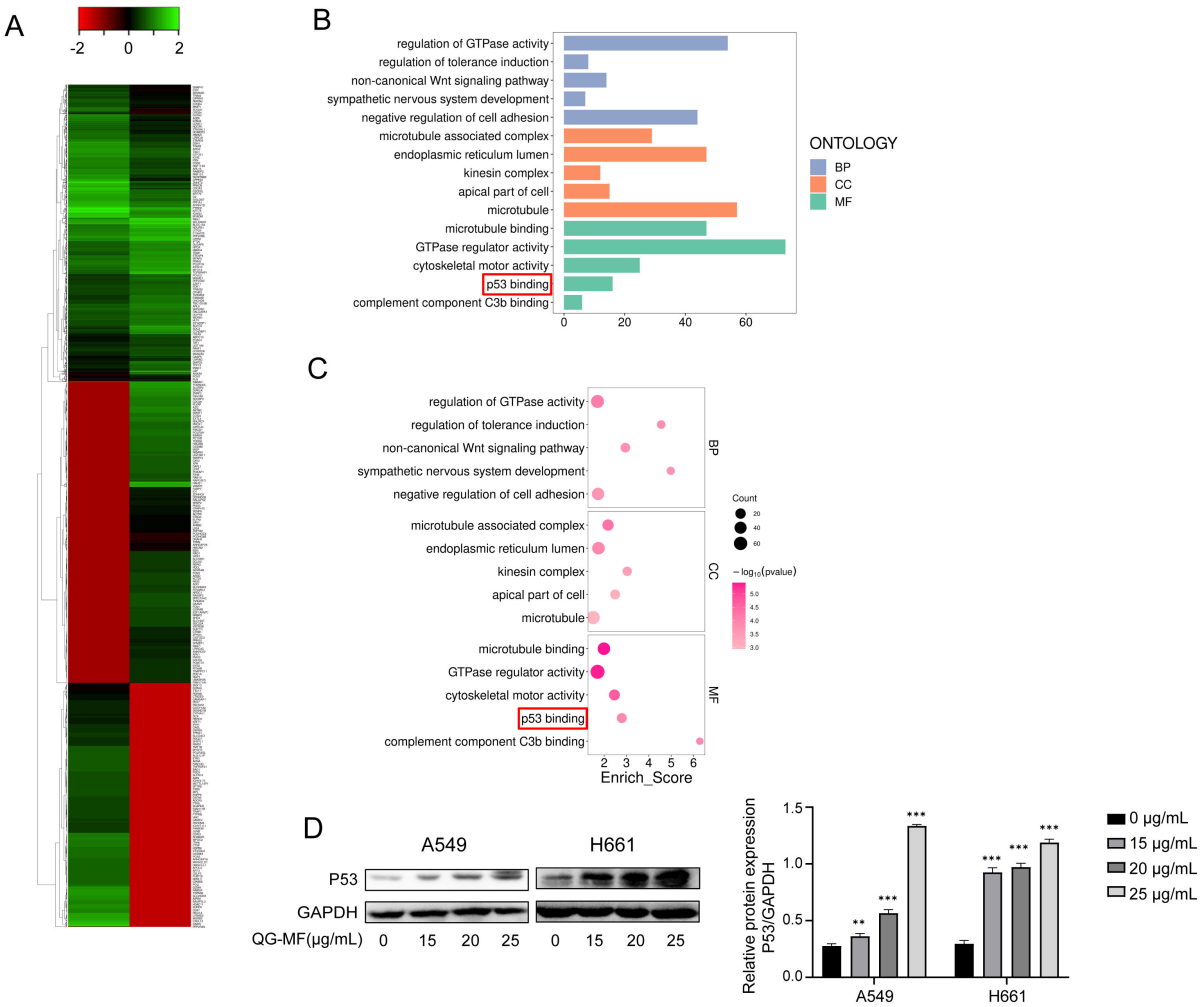
QG-MF up-regulated the expression of p53 in lung cancer cells. **(A)** Quantitative proteomic analysis of A549 cells treated with or without QG-MF. **(B, C)** Gene Ontology (GO) enrichment analysis of up-regulated proteins from QG-MF-treated versus untreated A549 cells. **(D)** Western blot analysis of p53 expression in A549 and H661 cells treated with the indicated concentrations of QG-MF for 48 hours. **p < 0.01, ***p < 0.001.

To investigate the mechanisms of QG-MF in lung cancer, we performed quantitative proteomics using UltraDeep diaPASEF (DIA) technology. Comparative analysis revealed 1,074 up-regulated and 1,326 down-regulated proteins in QG-MF-treated versus untreated A549 cells (**Figure 3A**). GO enrichment analysis demonstrated that the up-regulated proteins were significantly associated with the regulation of GTPase activity, microtubule-associated complexes, and p53 binding (**Figures 3B, C**). Given that the tumor suppressor p53 mediates cell cycle arrest and apoptosis in response to various anticancer drugs (20), we validated its expression. Consistent with our proteomic data, immunoblotting confirmed that QG-MF treatment up-regulated p53 protein levels in both A549 and H661 cells after 48 hours (**Figure 3D**).

### 3.4 P53 mediated the effect of QG-MF on lung cancer cells

**Figure 4:**
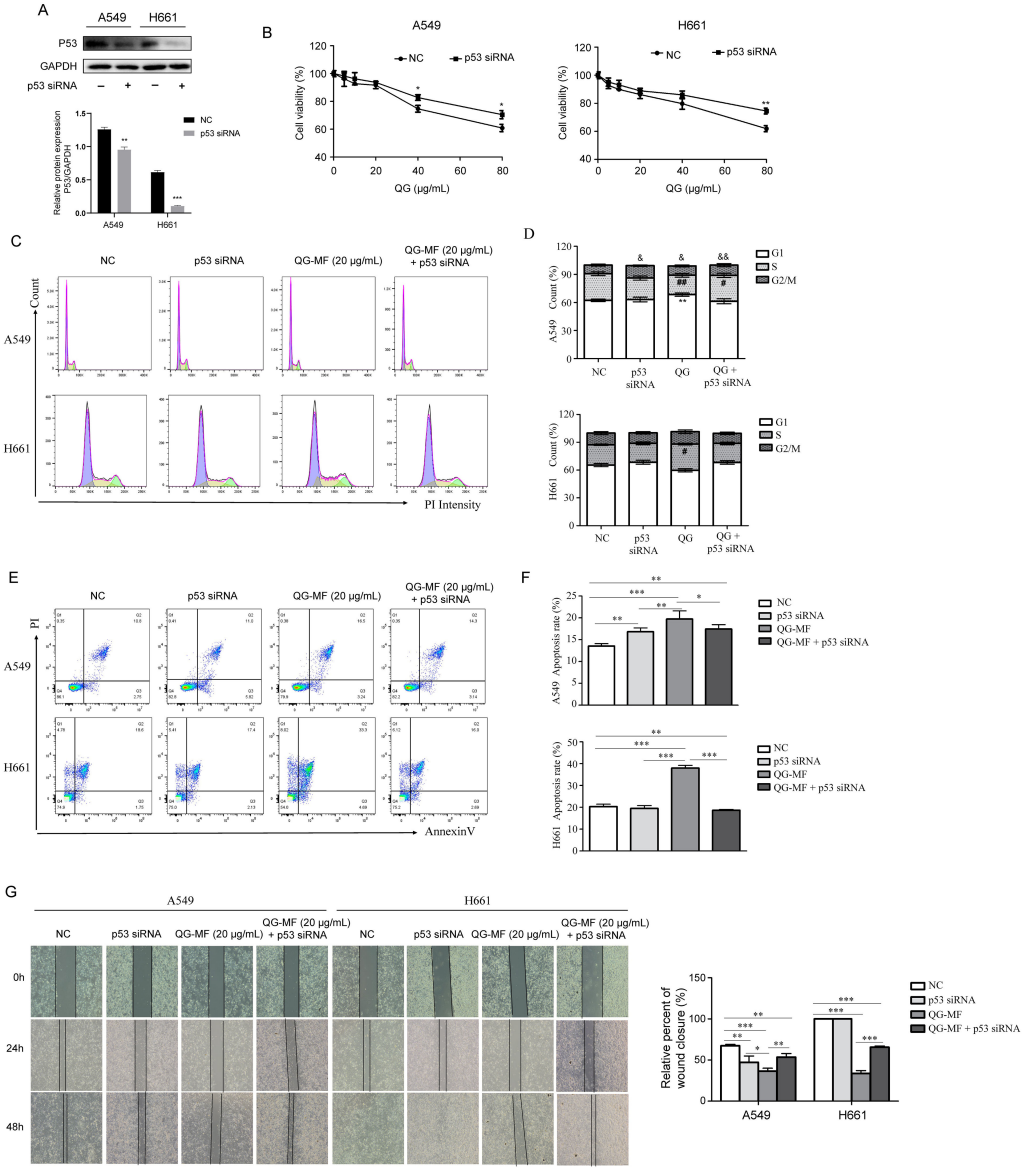
QG-MF inhibits proliferation, cell cycle progression, migration and induces apoptosis in lung cancer cells in a p53-dependent manner. **(A)** Validation of p53 knockdown by Western blotting. Cells were transfected with control siRNA (NC) or p53-specific siRNA (si-p53). **(B)** Cell viability was assessed by CCK-8 assay. After transfection with NC or si-p53 for 24 h, A549 and H661 cells were treated with QG-MF (0, 5, 10, 20, 40, 80 μg/mL) for 48 h. **(C, D)** Cell cycle distribution was analyzed by flow cytometry with PI staining. Cells were transfected and treated as in **(C)**, with a fixed QG-MF concentration of 20 μg/mL. **(E, F)** Cell apoptosis was analyzed by flow cytometry following Annexin V-FITC/PI staining. Cells were treated as described in **(D)**. **(G)** Cell migration ability was evaluated by wound healing assay. Representative images were captured at 0, 24, and 48 h after wounding, and the relative migration distance was quantified. Data are presented as the mean ± SD from three independent experiments. *p < 0.05, **p < 0.01, ***p < 0.001 compared between the indicated groups.

To determine whether p53 plays a critical role in the antitumor activity of QG-MF, we knocked down its expression using specific siRNA, with negative control (NC) siRNA as the control group (**Figure 4A**). p53 silencing significantly increased cell viability compared to the QG-MF-only treatment group (**Figure 4B**). Furthermore, the QG-MF-induced cell cycle arrest and apoptosis were markedly attenuated by p53 knockdown (**Figures 4C–F**). The migration abilities of A549 cells were also suppressed by p53 siRNA (**Figure 4G**). Taken together, these results indicate that p53 is a key mediator essential for QG-MF to exert its anti-tumor effects in lung cancer cells.

### 3.5 QG-MF activated p53/p21 pathway in lung cancer cells

**Figure 5:**
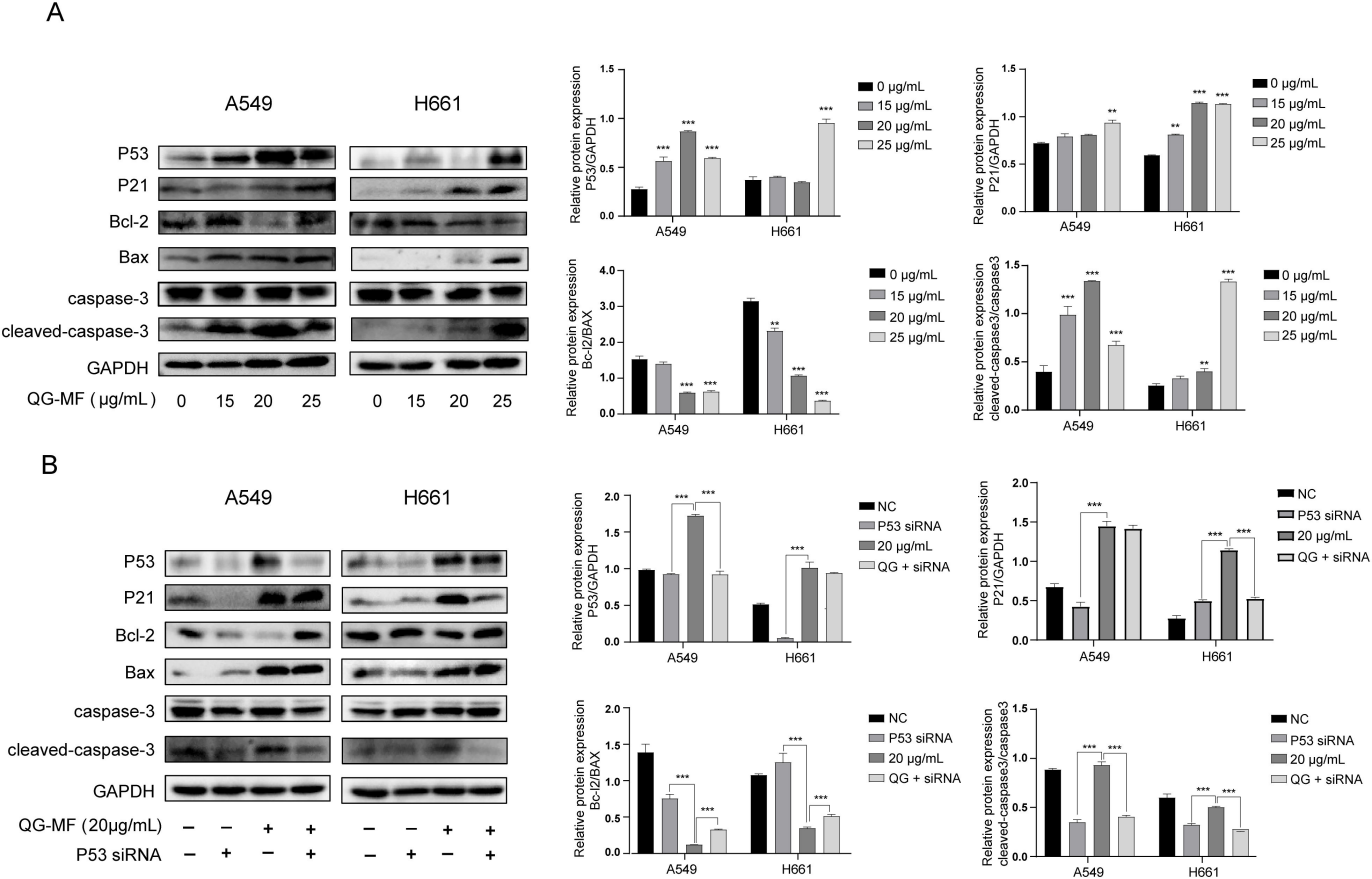
QG-MF upregulates the p53/p21 signaling pathway in lung cancer cells. **(A)** Western blot analysis of p53 and p21 protein expression in A549 and H661 cells after treatment with increasing concentrations of QG-MF for 48 h. **(B)** Western blot analysis of p53 and p21 expression in cells transfected with control siRNA (NC) or p53-specific siRNA (si-p53), followed by treatment with or without QG-MF for 48 h. **p < 0.01, ***p < 0.001.

The p53/p21 axis is a core signaling pathway that regulates cell cycle arrest. As a key downstream target of p53, p21 plays a central role in this process. Meanwhile, proteins such as the pro-apoptotic effectors Bax and caspase-3 promote cell death, whereas the anti-apoptotic protein Bcl-2 inhibits it (21). To determine if QG-MF modulates this pathway, we analyzed the expression of key regulatory proteins by Western blotting. The results showed that QG-MF treatment up-regulated the pro-apoptotic proteins (cleaved caspase-3 and Bax) and down-regulated the anti-apoptotic protein Bcl-2 (**Figure 5A**). Critically, when p53 expression was inhibited, these effects of QG-MF were significantly attenuated (**Figure 5B**). Collectively, these findings demonstrate that QG-MF triggers apoptosis and cell cycle arrest primarily through activation of the p53/p21 signaling pathway.

### 3.6 QG-MF inhibited tumor growth *in vivo*

**Figure 6:**
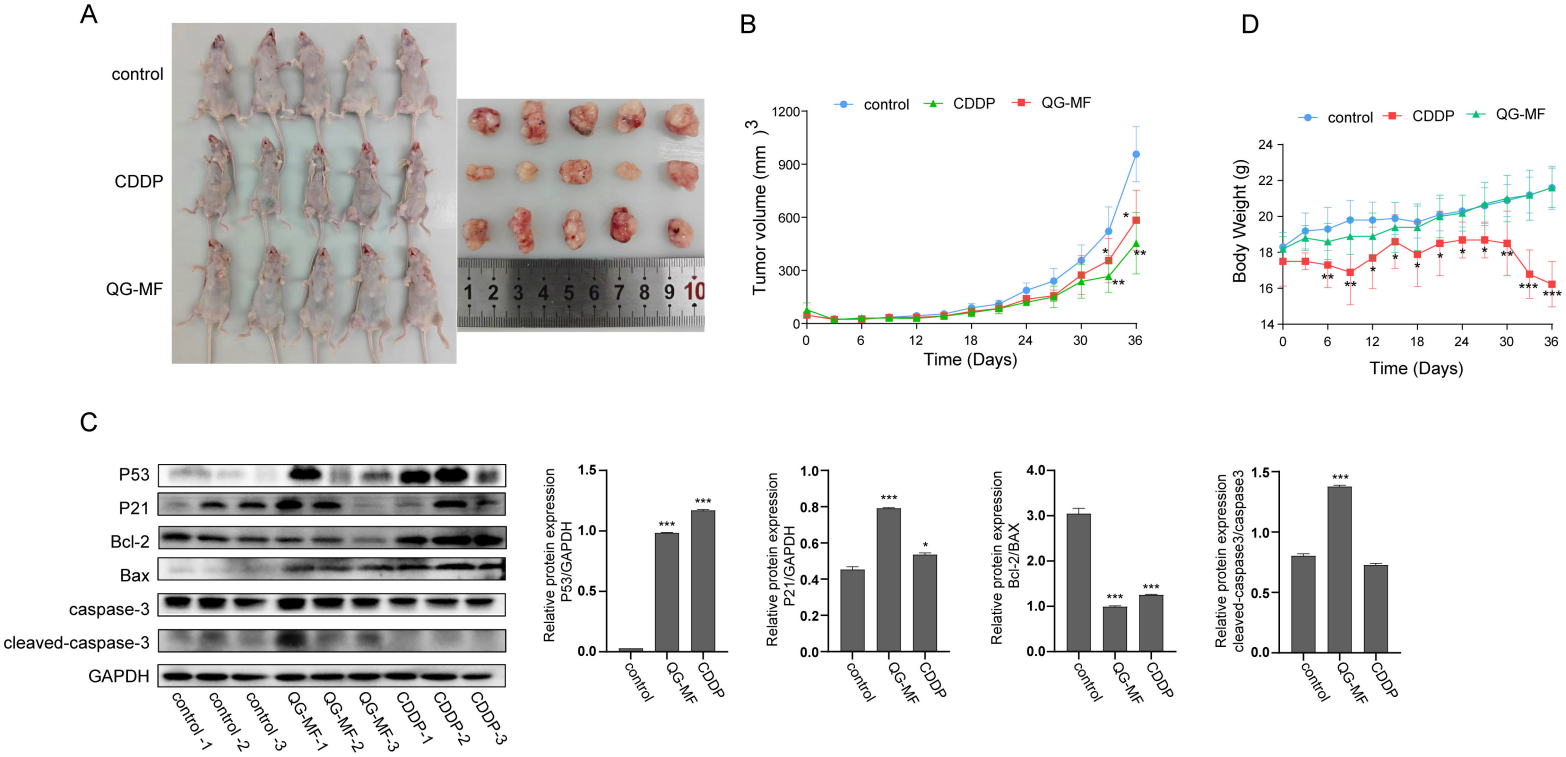
QG-MF suppresses tumor growth in xenograft models without significant toxicity. A549 cells (3 × 10^6^) were subcutaneously inoculated into the right flank of female BALB/c nude mice (n=5 per group). When tumor volumes reached approximately 60 mm^3^, mice were randomly assigned to three groups and treated for 36 days with: PBS, QG-MF, or cisplatin. **(A)** Representative images of excised tumors from each group at the endpoint of the study. **(B)** Tumor growth curves were plotted based on volume measurements taken every three days. **(C)** Body weight changes of mice were monitored every three days throughout the treatment period. **(D)** The protein expression in tumor tissues was detected by Western blot. Data in **(B, C, E)** are presented as the mean ± SD (n=6). *P < 0.05, **P < 0.01, ***P < 0.001 compared to the PBS control group.

Based on the *in vitro* growth-inhibitory effect of QG-MF on lung cancer cells (**Figure 1**), we evaluated its efficacy in an A549 xenograft mouse model. When tumor volumes reached approximately 60 mm^3^, mice were randomized into control, QG-MF, and cisplatin (CDDP) groups (n=5). QG-MF treatment significantly inhibited tumor growth compared to the control (p < 0.01), with efficacy comparable to CDDP (**Figures 6A, B**).

Mechanistically, analysis of tumor tissues revealed that QG-MF activated the p53/p21 pathway, evidenced by increased protein levels of p53 and p21. Concordantly, QG-MF treatment promoted apoptosis by upregulation of Bax and caspase-3; and concurrent downregulation of Bcl-2 (**Figure 6C**).

Regarding safety, mice treated with QG-MF maintained stable body weight throughout the study. In contrast, CDDP treatment caused a significant reduction in body weight from day 6 onward (p < 0.01) (**Figure 6D**). Serum biochemical analysis indicated the absence of hepatorenal toxicity with QG-MF treatment, as the relevant markers (ALT, AST, BUN, UA, CREA) showed no significant changes compared to the control group. In contrast, most of these parameters were significantly elevated in the CDDP group (**Table 1**). These findings collectively demonstrate that QG-MF effectively inhibits tumor growth *in vivo* while exhibiting a more favorable safety profile than cisplatin.

**Table 1:** Effects of QG-MF and cisplatin on serum biochemical parameters related to hepatorenal function.

Group	ALT(U/L)	AST(U/L)	BUN(mmol/L)	UA(μmol/L)	CRE(μmol/L)
Control	28.7 ± 2.7	143.0 ± 21.2	9.0 ± 1.0	161.1 ± 18.7	19.4 ± 2.8
CDDP	47.4 ± 9.5***	207.1 ± 63.6*	13.8 ± 2.8**	263.9 ± 50.4***	25.5 ± 2.5**
QG-MF	26.8 ± 1.6	89.6 ± 3.7	9.0 ± 0.7	174.7 ± 6.5	18.8 ± 2.3

Data are presented as mean ± SD (n=5).

* p < 0.05, ** p < 0.01, *** p < 0.001 vs. Control group.

## 4 Discussion

The present study systematically investigated the anti-tumor effects of quercetagetin-rich total flavonoids (QG-MF), extracted from the *Tagetes erecta* L. (marigold) lutein-extracted inflorescence residue, on NSCLC both *in vitro* and *in vivo*. Current findings demonstrate that QG-MF significantly suppresses NSCLC cell proliferation, migration, and invasion, while inducing cell cycle arrest and apoptosis, and these effects are mediated by the activation of the p53/p21 signaling pathway. This work not only elucidates the molecular mechanism of QG-MF against NSCLC but also highlights the potential of marigold lutein residues as a high-value source for natural anti-cancer agents, aligning with the global trend of sustainable utilization of plant by-products.

Natural flavonoids derived from medicinal plants have long been recognized as promising anti-tumor candidates due to their diverse biological activities and low toxicity, and marigold (*Tagetes erecta* L.) has been extensively studied for its flavonoid-rich composition in ethnomedicinal practices (22). Quercetagetin, the major flavonol component of marigold extracts, has been reported to inhibit the progression of multiple malignancies, including lung cancer, colon cancer, and melanoma (23–25). Notably, this study focuses on QG-MF — a quercetagetin-enriched total flavonoid fraction (85% quercetagetin, 94% total flavonoid purity) from marigold lutein residues — rather than isolated quercetagetin monomer. Such synergism can enhance therapeutic efficacy and reduce potential toxicity, a phenomenon widely observed in flavonoid-rich plant extracts (26). Acquired results confirm that QG-MF inhibits NSCLC growth in a dose-dependent manner, extending previous findings on marigold flavonoids by linking their activity to a specific signaling pathway and validating their efficacy in both cellular and animal models.

Dysregulated cell cycle progression and impaired apoptosis are hallmarks of NSCLC, making these processes key targets for anti-cancer drug development (27, 28). Obtained data show that QG-MF induces cell cycle arrest and promotes apoptosis in A549 and H661 cells, which is mechanistically associated with the activation of the p53/p21 signaling axis — a well-characterized tumor suppressor pathway. As a central regulator of genomic stability, p53 exerts anti-tumor effects by transcriptionally activating pro-apoptotic genes (e.g., Bax) and cell cycle regulatory genes (e.g., p21) (29, 30). p21, in turn, inhibits cyclin-CDK complexes to block cell cycle progression and enhances apoptosis, forming a functional cascade with p53 (31–33). Consistent with this, QG-MF was found to upregulate the expression of p53, p21, and Bax, while downregulating the anti-apoptotic protein Bcl-2 — a pattern that is conserved in both *in vitro* and *in vivo* models. Furthermore, siRNA-mediated p53 knockdown significantly attenuated QG-MF’s anti-tumor effects, confirming that the p53/p21 pathway is indispensable for QG-MF’s efficacy. This mechanism is consistent with previous reports on flavonoid-induced anti-tumor activity: for instance, Li et al. demonstrated that epiberberine induces G2/M arrest and apoptosis in gastric cancer via p53 accumulation and p21/CDK1/cyclin B1 pathway activation (34). The present study extends this paradigm to marigold-derived total flavonoids, highlighting the conserved role of the p53/p21 pathway in flavonoid-mediated anti-NSCLC activity.

Although p53 silencing greatly weakened the therapeutic effects of QG-MF, residual anti-tumor activity was still detectable (**Figure 4**). This indicates that QG-MF exerts its anticancer actions mainly via the p53/p21 cascade, and meanwhile, additional p53-independent signaling pathways are also involved. Apart from the above pathways, the NF-κB cascade is well documented to participate in the modulation of cell cycle progression and apoptosis in tumor cells. Aberrant activation of NF-κB generally promotes malignant proliferation and inhibits cell death, and mutual crosstalk exists between NF-κB and the p53/p21 signaling axis. It is speculated that QG-MF may also exert anti-NSCLC activity via regulating the NF-κB pathway. Further research will focus on exploring the interaction between these two pathways and clarifying their combined effects. As plant-derived flavonoids generally act on multiple molecular targets, QG-MF may regulate other pathways such as MAPK and PI3K/Akt to mediate cell cycle arrest and apoptosis. Further investigations will be conducted to explore these potential alternative mechanisms.

Notably, QG-MF triggered cell cycle arrest at different phases in A549 and H661 cells. This difference is closely related to their disparate p53 status. A549 cells express wild-type p53, which predominantly activates the G1 checkpoint and leads to G1-phase arrest after QG-MF treatment. As H661 cells are p53-deficient, the G1 checkpoint function is impaired, so the cells are arrested at other cell cycle phases instead. Moreover, the two cell lines differ in the endogenous expression of cyclins and CDKs, which further contributes to their divergent cell cycle responses to QG-MF.

We noticed a minor deviation in the expression of p53 in H661 cells and p21 in A549 cells upon QG-MF treatment (**Figure 5B**). This discrepancy is primarily attributed to the distinct genetic backgrounds of the two NSCLC cell lines. A549 cells carry wild-type p53, while H661 cells are p53-deficient, which leads to a blunted p53 response to drug intervention. In addition, p21 expression is regulated by both p53-dependent and p53-independent signaling pathways, such as E2F and TGF-β cascades. These alternative regulatory mechanisms may uncouple the expression trends of p53 and p21 in A549 cells. Such subtle variations are commonly observed in cellular pharmacological experiments and do not alter the core conclusion that QG-MF executes its anti-tumor function mainly through the p53/p21 axis.

A key innovation of this study lies in the valorization of marigold lutein residues — a major by-product of industrial lutein extraction that is often discarded as waste, causing resource loss and environmental burden. Focus on lutein residues not only reduces production costs but also enhances the environmental and economic value of Tagetes erecta L. Despite these promising findings, several limitations of the present study should be acknowledged. First, although QG-MF is identified as a quercetagetin-enriched flavonoid fraction, individual flavonoid components were not isolated, and the potential synergistic effects among different constituents remain unclear. Future work will purify single compounds from QG-MF to clarify whether the anti-NSCLC activity is derived from quercetagetin alone or from synergistic interactions of multiple flavonoids. Second, although serum biochemical indicators indicated no obvious liver and renal toxicity, systematic histological evaluation of tumor and major visceral tissues was not performed, which limits the comprehensive assessment of *in vivo* safety. Further histopathological staining will be conducted to verify the morphological changes of tissues and exclude potential systemic toxicity. Third, the current study only validated the regulatory effects of QG-MF on the p53/p21 pathway at the protein expression level. Direct transcriptional evidence, such as ChIP assay to confirm the binding of p53 to p21 and Bax promoters, is still required to further solidify the pathway activation mechanism. Fourth, the tumor selectivity of QG-MF was not evaluated; cytotoxicity assessment using normal lung epithelial cells (e.g., BEAS-2B) will be performed in future studies to confirm its safety and selective anti-cancer property. Finally, pharmacokinetic profiles, including oral bioavailability, absorption, distribution, metabolism, and excretion, remain uncharacterized in this work. Further pharmacokinetic investigation is necessary to optimize oral dosage regimens and support the future translational application of QG-MF.

In conclusion, current evidence demonstrates that QG-MF exerts potent anti-NSCLC activity by activating the p53/p21 signaling pathway, thereby inducing cell cycle arrest and apoptosis and inhibiting tumor metastasis. This work not only provides experimental evidence for the ethnomedicinal application of *Tagetes erecta* L. but also validates the potential of marigold lutein residues as a sustainable source for natural anti-cancer agents. These findings support QG-MF as a promising candidate for further development into novel NSCLC therapeutics and highlight the value of repurposing agricultural byproducts for pharmaceutical research.

## Data Availability

The raw proteomics data generated in this study have been deposited to the iProX (35, 36) database (https://www.iprox.cn/) under project IDs IPX0017768000 (Label-free profiling) and IPX0017774000 (DIA quantitative profiling). The official ProteomeXchange accession numbers will be available after dataset review, and the final public dataset link will be updated upon manuscript publication.
